# Multidirectional associations between the gut microbiota and Parkinson’s disease, updated information from the perspectives of humoral pathway, cellular immune pathway and neuronal pathway

**DOI:** 10.3389/fcimb.2023.1296713

**Published:** 2023-12-15

**Authors:** Xiaokang Jia, Qiliang Chen, Yuanyuan Zhang, Tetsuya Asakawa

**Affiliations:** ^1^ School of Traditional Chinese Medicine, Hainan Medical University, Haikou, Hainan, China; ^2^ School of Basic Medicine, Guangzhou University of Chinese Medicine, Guangzhou, Guangdong, China; ^3^ Department of Acupuncture and Moxibustion, The Affiliated Traditional Chinese Medicine (TCM) Hospital of Guangzhou Medical University, Guangzhou, Guangdong, China; ^4^ Institute of Neurology, National Clinical Research Center for Infectious Diseases, the Third People’s Hospital of Shenzhen, Shenzhen, Guangdong, China

**Keywords:** gut microbiota, Parkinson’s disease, microbiota-gut-brain axis, inflammatory reaction, neuronal pathway

## Abstract

The human gastrointestinal tract is inhabited by a diverse range of microorganisms, collectively known as the gut microbiota, which form a vast and complex ecosystem. It has been reported that the microbiota-gut-brain axis plays a crucial role in regulating host neuroprotective function. Studies have shown that patients with Parkinson’s disease (PD) have dysbiosis of the gut microbiota, and experiments involving germ-free mice and fecal microbiota transplantation from PD patients have revealed the pathogenic role of the gut microbiota in PD. Interventions targeting the gut microbiota in PD, including the use of prebiotics, probiotics, and fecal microbiota transplantation, have also shown efficacy in treating PD. However, the causal relationship between the gut microbiota and Parkinson’s disease remains intricate. This study reviewed the association between the microbiota-gut-brain axis and PD from the perspectives of humoral pathway, cellular immune pathway and neuronal pathway. We found that the interactions among gut microbiota and PD are very complex, which should be “multidirectional”, rather than conventionally regarded “bidirectional”. To realize application of the gut microbiota-related mechanisms in the clinical setting, we propose several problems which should be addressed in the future study.

## 1 Introduction

Parkinson’s disease (PD) is the leading movement disorders and the second leading neurodegenerative disease (NDD), with which patients are expected to be over doubled in the next 20 years (Asakawa et al., 2016b; Qin et al., 2022). The activity of daily living (ADL) and quality of life (QOL) in the advanced patients are significantly affected, however, more officious treatments are under developing. Till now, dopaminergic medicine and surgical therapy like deep brain stimulation (DBS) remain the mainstay treatments against PD (Qin et al., 2022). There has been no revolutionary breakthrough in terms of new therapy since 1967 levodopa and 1993 DBS emerged. It is difficult to obtain a novel but satisfactory treatment against PD because of the extreme complex and multifold pathophysiological mechanisms of PD, which remains unclear by far (Qin et al., 2022). Hence, clarifying the PD related mechanisms has been an urgent task faced by the global PD researchers. Although many researchers have attempted to interpret PD related mechanisms from multidimensions, the substantial pathogenesis (we call it “neurotoxic environment”) to cause apoptosis of the dopaminergic neurons in the substantial nigra remains intricate. Aging related neurodegeneration seems to be a reasonable interpretation for many PD related changes. However, the factor of aging cannot elucidate those patients with PD onset in a young age. Many animal models were established according to the known neurotoxins. Unfortunately, none of these models can completely imitate the human’ PD (Asakawa et al., 2016a), which implies that neurotoxin might not be a main cause of this “neurotoxic environment”. It seems to indicate that the “neurotoxic environment” is made from many factors. PD is the final comprehensive result of the complicated interaction/crosstalk among these factors. Other than the conventional environmental and genetic pathogenic factors, recently, the roles of gut microbiota and the related microbiota-gut-brain axis come into the picture, particularly with the development of the high-throughput sequencing (Tan et al., 2022).

The relationship between gastrointestinal (GI) dysfunction and PD was noticed even by James Parkinson, who first reported PD in 1817 (Tan et al., 2022). It is reported that GI dysfunction is consistently found in 80% early PD patients (Fasano et al., 2015; Killinger et al., 2018). Gut microbiota represents a huge community of bacteria residing in the GI tract (Jia et al., 2021). The total amount of gut microbiota genes is approximately 100 times than that of human genes and is known as the “second genome” of humans (Moeller et al., 2012; Komaroff, 2018). Using the technology like high-throughput sequencing, investigation regarding the microbiota-gut-brain axis stepped into a new era. Recently, a growing number of studies have elucidated the PD-related metabolic changes of gut microbiota are closely associated with the occurrence and development of PD (Shen et al., 2021; Tan et al., 2021). These “impaired” gut microbiota along with their relevant metabolites are believed to interact with the host, mainly via the microbiota-gut-brain axis, and finally become a pathogenic factor of PD (Mayer et al., 2015). Additionally, many later studies suggested that gut microbiota can be considered as the diagnostic/therapeutic biomarkers for PD, or biomarkers to display the PD progression (Schapira, 2013; Neurology, 2016; Nair et al., 2018). However, due to the characteristics of gut microbiota, somewhile it is difficult to make a directed interaction to change the behaviors of the gut microbiota. Hence, development of a medication against PD on the basis of the gut microbiota related mechanisms are quite difficult. Recently, Tan and her colleagues published an insightful study discussing the available evidence regarding the microbiome–gut–brain axis and PD (Tan et al., 2022). This study critically reviewed the complex interaction/crosstalk between the brain and gut via the microbiome–gut–brain axis. Based on this study, we motivated this review focusing on the association between gut microbiota and PD from the perspectives of humoral pathway, cellular immune pathway and neuronal pathway. We attempted to provide the wider and update information on this topic. We believe these take-home- messages will deepen the understanding the roles of gut microbiota in the PD scenario, and will be helpful for developing novel diagnostic/therapeutic targets against PD based on the gut microbiota.

## 2 Pathological links between PD and Gut microbiota

Clinically, many PD patients suffer from GI dysfunction, this might be the first noteworthy symptoms which drew the attention of the clinicians to investigate the role of GI in PD. Indeed, whether it is the GI dysfunction causes PD, or conversely PD causes GI dysfunction is a chicken-and-egg problem. Although growing evidence elucidated that dysbiosis of gut microbiota plays a pathogenic role in development of PD, where the microbiota-gut-brain axis plays a key role, nowadays, the mainstream opinion is that the association between PD and Gut microbiota is bidirectional (Tan et al., 2022): Certainly, dysbiosis of gut microbiota may lead PD development, and GI dysfunction may be a non-motor symptom in PD. Importantly, dysbiosis of gut microbiota exhibits complicated interactions with PD pathophysiology, and comprehensively drives the PD progress.

### 2.1 Dysbiosis of gut microbiota in PD subjects

PD patients may experience many symptoms of GI dysfunction, such as constipation, delayed gastric emptying, altered bowel habits, and nausea (Marrinan et al., 2014; Pellegrini et al., 2015; Mrabet et al., 2016). Indeed, these symptoms have been noticed as non-motor symptoms in PD. It has been reported that PD patients are commonly accompanied with and infection of *Helicobacter pylori* infection and peptic ulcer (Rees et al., 2011; Tan et al., 2020). Interestingly, when the patient-derived α-synuclein (α-syn) were stereotactically injected into the striatum or enteric nerves in a non-human primate animal, both injections would cause lesions in the nigrostriatal pathway and enteric nervous system (Arotcarena et al., 2020). This finding indicates that exposure of α-syn may results in pathological changes, not only in brain, but also in GI. Numerous studies revealed the abnormality of the gut microbiota in PD. Structure and function of the gut microbiota in PD patients are quite different in comparison with the healthy subjects among these studies (**Table 1**). The heterogeneous results indicate that the changes of gut microbiota are affects by many complicated factors such as age, geographic provenance, dietary habit, research protocols and sequencing methods (Maier et al., 2018; Boertien et al., 2019). Of those, *Akkermansia* was reportedly increased in PD patients (Gerhardt and Mohajeri, 2018; Baldini et al., 2020; Nishiwaki et al., 2020; Vascellari et al., 2020; Zhang et al., 2020). Meanwhile, abundance of *Bifidobacterium* was reportedly increased (Vascellari et al., 2020; Li et al., 2021; Tan et al., 2021; Yan et al., 2021b). *Roseburia* and *Prevotella* were observed to be significantly decreased in PD patients (Nishiwaki et al., 2020; Vascellari et al., 2020; Zhang et al., 2020; Li et al., 2021), and a decrease in *Prevotella* was observed, which was negatively correlated with PD pathogenesis (Hasegawa et al., 2015). Additionally, many studies indicated close association between dysbiosis of gut microbiota and PD-related clinical manifestations like PD severity, duration, motor and nonmotor symptoms (Ren et al., 2020; Zheng et al., 2021).

**Table 1:** Microbial alterations in patients with PD.

Studies	Country	Participants and intervention	Changes in intestinal microflora		Detection methods
			Decreased	Increased	
Tan et al., 2021	Malaysia	Patients with PD (n=104), together with spouse (n=91) or sibling (n=5) controls free of neurological disorders and living in the same community	Not listed	*Cloacibacillus*, *Catabacter*, *Christensenella*, *Butyrivibrio*, and *Bifidobacterium*	16S rRNA
Qian et al., 2020	China	Patients with PD [37 (47.4%) female; age 67.0 (5.6) years], 75 healthy control subjects [36 (48.0%) female; age 65.3 (7.5) years]	Not listed	King-doms Viruses and Archaea and of the phyla Synergistetes, Verrucomicrobia and Viruses with no name	Shotgun metagenomic sequencing
Yan et al., 2021b	China	Patients with PD (n=20); healthy control subjects (n=20)	*Faecalibacterium*	Alistipes, Rikenellaceae_RC9_gut_group, *Bifidobacterium*, *Parabacteroides*	16S rRNA
Vascellari et al., 2020	Italy	Patients with PD (n=64) and healthy controls (n=51)	*Bacteroides, Blautia, Lachnospira, Butyrivibrio, Roseburia, Pseudobutyrivibrio, Brevibacterium, Dolichospermum, Coprococcus, and Odoribacter.*	*Akkermansia*, *Escherichia*, *Bifidobacterium*, *Streptococcus*, *Clostridium*, *Serratia*, *Veillonella*, *Prosthecobacter*, *Enterobacter, and Slackia*.	16S rRNA
Nishiwaki et al., 2020	Japan	Patients with PD (n=223); healthy control subjects (n=137)	*Faecalibacterium*, *Roseburia, and Lachnospiraceae* ND3007 group	*Genus Akkermansia and family Akkermansiaceae*	16S rRNA
Baldini et al., 2020	Luxembourg	Patients with PD (n=147); healthy control subjects (n=162)	*Paraprevotella*	*Akkermansia、Christensenella and Lactobacillus*	16S rRNA
Li et al., 2021	China	Patients with PD (n=69) and controls in three groups ((n=244, comprising 66 spouses, 97 age-matched, and 81 normal samples, respectively)	*Roseburia*	c_Actinobacteria, f_Bifidobacteriaceae, g_Bifidobacterium, o_Bifidobacteriales, p_Actinobacteria f_Lactobacillaceae, and *g_Lactobacillus*	Shotgun metagenomic sequencing
Zhang et al., 2020	China	Patients with PD (n=63), healthy spouses (HS) (n=63)and healthy people (n=74)	*Fusobacterium*, *Prevotella*	*Oscillospira*, *Akkermansia*	16S rRNA

Put another way, dysbiosis of gut microbiota is also observed in PD animal models (**Table 2**), albeit all the animal models available nowadays cannot completely imitate the pathophysiological changes in PD patients (Asakawa et al., 2016a). Similar to PD patients, *Akkermansia* was also increased (Gorecki et al., 2019; Yan et al., 2021a; Zhao et al., 2021), whereas abundance of *Prevotella* was reportedly decrease in these animal models (Sampson et al., 2016; Joers et al., 2020; Yan et al., 2021a). Similar to the scenario in Human patients, the changes of gut microbiota in animal models are also influenced by many factors such as neurotoxin used, and administrating approaches, dose, and timing.

**Table 2:** Microbial alterations in animal PD models.

Studies	Country	Animal models	Changes in intestinal microflora		Detection methods
			Decreased	Increased	
Yan et al., 2021a	China	A53T transgenic monkeys with early PD symptoms	*Prevotella*	*Sybergistetes*, *Akkermansia*, *and Eggerthella lenta*	Shotgun metagenomic sequencing
Sun et al., 2018	China	MPTP-induced PD mice model	Phylum Firmicutes and order Clostridiales	Phylum Proteobacteria, order Turicibacterales and Enterobacteriales	16S rRNA
Zhao et al., 2021	China	MPTP-induced PD mice model	Bacteroidetes phylum	Genera *Akkermansia, Desulfovibrio,Verrucomicrobia* phylum and Verrucomicrobiaceae family	16S rRNA
Joers et al., 2020	USA	MPTP-induced PD monkey model	*Prevotella*	Firmicutes phylum	16S rRNA
Perez-Pardo et al., 2018	Netherlands.	Rotenone-induced PD mouse model	*Actinobacteria* and *Bifidobacterium*	Phyla Bacteroidetes and Firmicutes	16S rRNA
Gorecki et al., 2019	Australia	Human α-syn over-expressing PD mouse model	*Verrucomicrobiae*	*Akkermansia*	16S rRNA
Sampson et al., 2016	USA	Mice that overexpress α-syn	*Lachnospiracae, Rikenellae, Peptostreptococacae and Butyricicoccus *sp	*Proteus* sp.*, Bilophila* sp.*, and Roseburia sp*	16S rRNA
Lai et al., 2018	China	A neurotoxin PD mouse model induced by chronic low doses of MPTP	*Prevotellacea, Clostridiale*	*Erysipelotrichaceae*	16S rRNA

Some authors believed that specific gut microbiota might be considered as a potential marker and therapeutic target for PD because the changes of PD-related gut microbiota are closely related to the occurrence and development of PD (Ji et al., 2021). However, due to the heterogeneous nature of these results, no matter in human patients and in animal models, till now, there remains no compelling evidence supporting which gut microbiota can be served as a diagnostic/therapeutic target against PD. In addition, the aforementioned results are derived from investigations with small samples in different experimental conditions. Moreover, they cannot answer the chicken-and-egg problem, namely whether the pathophysiological changes of PD cause dysbiosis of gut microbiota, or *vice versa*. Hence, before we can conclude the roles of gut microbiota in PD, much more deep and rigorous investigations are desired.

### 2.2 The modulatory role of gut microbiota in neurological functions

The interaction/crosstalk between brain and gut microbiota is increasingly attended. Gut microbiota is living in a relatively balanced environment in a normal human, which may contribute to modulation of the neurological functions such as learning, cognitive and memory (Parashar and Udayabanu, 2017; Cryan et al., 2020). Cryan et al. reported that gut microbiota can regulate intellectual and behavioral development, and is associated with some neurological disorders (Cryan et al., 2019). A number of investigations recently documented that complete absence or severe depletion of the gut microbiota may result in impairments of learning and memory in the hosts (Chu et al., 2019; Yu et al., 2019; Liu et al., 2020). Olson et al. found that co-treatment of ketogenic diet and hypoxia can change the gut microbiota with *bilophila wadsworthia* enriched, then promote cognitive impairment in germfree (GF) mice (Olson et al., 2021). Wu et al. reported that enterococcus faecalis can affect social behavior through discrete neuronal circuits and mediate stress response in GF mice (Wu et al., 2021c).

In the scenario of PD, many studies also elucidated a close association between PD and gut microbiota. Sampson et al. reported that gut microbiota regulates motor deficits and neuroinflammation in α-syn overexpressed mice. Importantly, once the gut microbiota derived from PD patients was transplanted into these models, the neurological deficits were remarkably enhanced (Sampson et al., 2016). Wekerle reported that Escherichia coli and Salmonella typhimurium may induce secretion of the Curli protein and trigger accumulation of α-syn in aged Fischer 344 rats and Caenorhabditis elegans (Wekerle, 2017). Later, Sun et al. found that fecal microbiota transplantation (FMT) resulted in attenuation of microglial activation, decrease of short-chain fatty acids (SCFAs) and caused motor deficits (Sun et al., 2018). Clinically, it has been documented that gut microbiota can influence PD severity (Malkki, 2017; Rani and Mondal, 2021).

These aforementioned reports suggested a close link between PD and gut microbiota from multidimensions. However, direct evidence elucidating PD and gut microbiota remains insufficient. In this regard, it is indispensable to discuss the potential roles of several factors involved in the interactions between PD and gut microbiota.

### 2.3 Roles of microbiota-gut-brain axis

**Figure 1:**
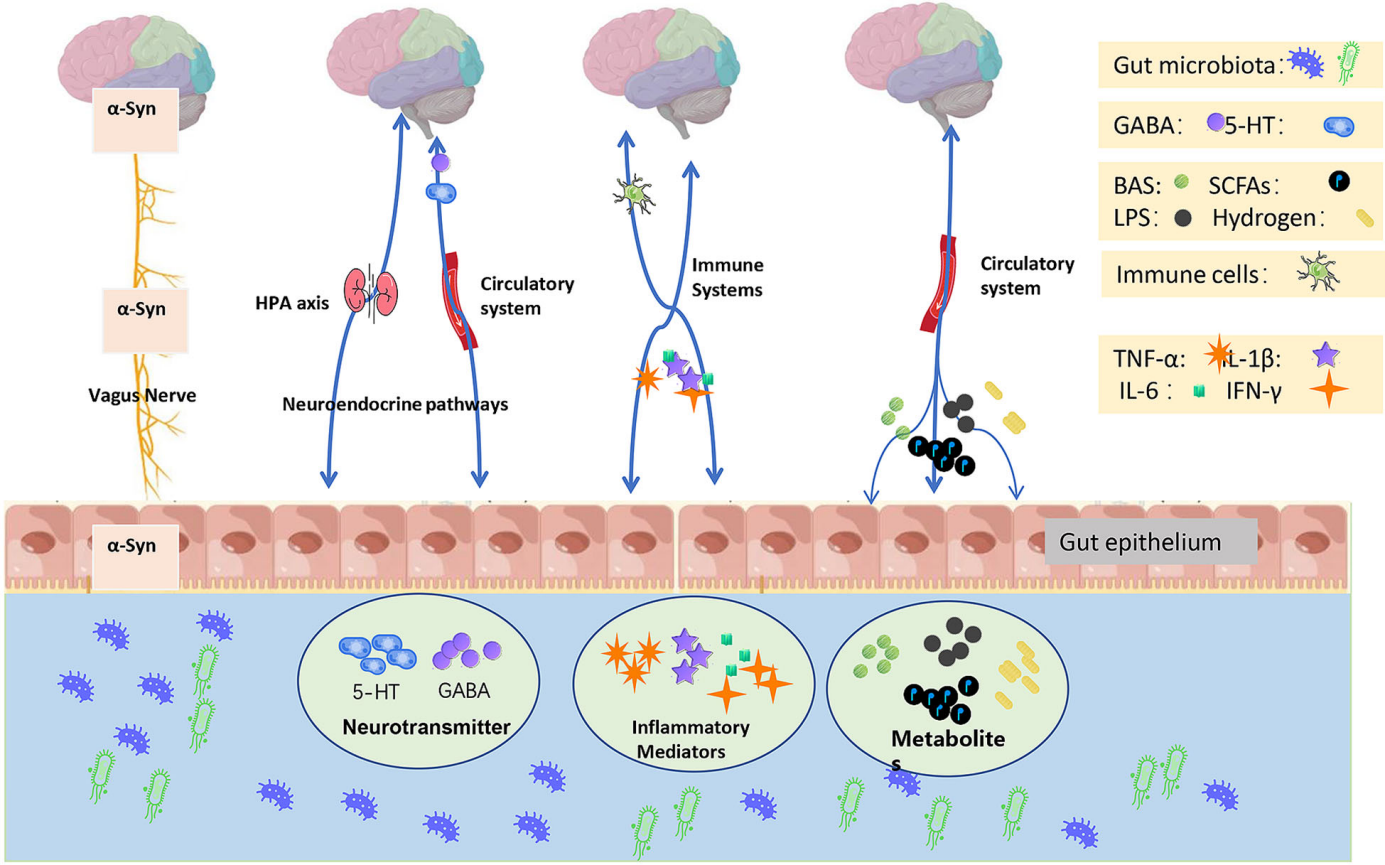
Microbiota-gut-brain axis-related PD pathology. Intestinal microbiota along with its derivatives play a role in the pathology of PD. The emerging mechanisms include modulation of the gut environment, maintenance of the ecological balance among the gut microbiota, and effects on the neurotransmitters, inflammatory factors, and related metabolites within the gut. The potential influence pathways were *i)* the vagus nerve conveys α-syn; *ii)* the neuroendocrine pathways primarily involve the HPA axis, and the circulation-mediated neurotransmitters such as serotonin (5-HT) and gamma-aminobutyric acid (GABA); *iii)* the immune system regulates immune cells and mediates the release of inflammatory factors; and *iv)* the circulation system transports BAS, SCFAs, LPS, and hydrogen.

Recently, Tan et al. reviewed the potential roles of microbiota-gut-brain in the PD scenario (Tan et al., 2022). Indeed, microbiota-gut-brain axis plays a key role in the interactions between gut microbiota and brain. It is known that at least four systems involved in the modulation of the microbiota-gut-brain axis, namely central nervous system (CNS), autonomic nervous system (ANS), hypothalamic pituitary adrenal (HPA) axis, and enteric nervous system (ENS) (Cryan et al., 2019; Heiss and Olofsson, 2019; Rutsch et al., 2020; Doroszkiewicz et al., 2021). Delivery of inflammatory signals plays a key role in the interaction between brain and gut microbiota (Agirman et al., 2021). A conventional theory is that dysbiosis of gut may induce peripheral inflammation, the inflammatory signals can be delivered into the brain via these pathways thereby cause the inflammatory response in CNS, ultimately form the “neurotoxic environment” in the brain, which might be a pathogenic factor causing PD. However, there is no direct evidence proving that dysbiosis of gut microbiota might cause PD. Several hypothetic pathways were introduced contributing the inflammatory interactions among brain, gut and microbiota, including humoral pathway, cellular immune pathway and neuronal pathway (Agirman et al., 2021). However, these pathways are also problematic in terms of PD pathophysiology (**Figure 1**).

#### 2.3.1 Humoral pathway

Agirman et al. introduced that dysbiosis of gut microbiota may trigger release of pro-inflammatory cytokines and disrupt the blood brain barrier (BBB) integrity, then make a gateway which may introduce peripheral toxins, and pathogens into brain and cause neuroinflammation (Agirman et al., 2021). Meanwhile, such neuroinflammation may trigger release of glucocorticoid and deteriorate the environment of gut microbiota. These bidirectional regulations create a vicious circle. However, this pathway is mainly based on the BBB damage. If the BBB is relative unbroken, the interactions between CNS and peripheral blood are limited. Albeit a recent report indicated that BBB damage was found in PD with dementia (Wong et al., 2022), there is no direct clinical evidence showing the BBB damage in PD without cognitive impairment, particularly in early stage of PD. Hence, other than the cytokines, liposoluble small molecules might play a more important role in the humoral pathway, such as metabolites in the tryptophan–kynurenine pathway (KP), SCFAs, and bile acids (BAs) (Qin et al., 2022).

##### 2.3.1.1 The roles of tryptophan metabolites

Tryptophan is an essential amino acid which approximately over 95% is metabolized through KP whereas less than 5% is generates 5-HT. Importantly, both metabolites in KP and 5-HT are proven to be closely associated with the PD pathophysiology.

###### 2.3.1.1.1 KP and PD

Recently, we reviewed the role of KP in the pathogenicity of PD (Qin et al., 2022). Both aging and dysbiosis of gut microbiota may activate some rate-limiting enzymes in KP, such as indoleamine 2,3-dioxygenase 1 (IDO1) and tryptophan 2,3-dioxygenase (TDO). Activation of KP will result in enhancement of the downstream toxins, such as 3-hydroxykynurenine, 3-hydroxyanthranilic acid quinolinic acid. Although these toxins are mainly produced peripherally, the liposoluble nature with small molecular weight make they are easily cross the BBB and accumulate in the CNS, thereby exhibiting neurotoxic effects. Hence, at the state of BBB remains unbroken, KP may act as an “aging signal deliverer”, particularly deliver inflammatory signal from the periphery to the CNS. Close association among KP, α-syn, and PD has been well documented at this review (Qin et al., 2022). However, there remains no direct evidence supporting the activation of KP will cause α-syn deposition and ultimately cause PD (Qin et al., 2022).

###### 2.3.1.1.2 The roles of 5-HT

As a minor production of the tryptophan metabolism, 5-HT cannot directly cross BBB. Hence, the 5-HT in CNS is generated in the CNS in case of BBB is unbroken. Many studies reported the close association between 5-HT and gut microbiota. It is documented that the hippocampal 5-HT and its’ main catabolic product levels increase whereas plasma tryptophan, and tryptophan metabolism via KP decrease in the GF mice (Wikoff et al., 2009; Diaz Heijtz et al., 2011). Animal experiments demonstrated that after administration of antibiotics in GF mice, the levels of 5-HT in colon and feces decreased, nevertheless reconstruction of gut microbiota restored 5-HT levels in these animals (Yano et al., 2015; Ge et al., 2017). Desbonnet et al. found that *Bifidobacterium infantis* can increase plasma tryptophan level, which may influence central 5-HT transmission (Desbonnet et al., 2010). These results imply that 5-HT is remarkably affected by the gut microbiota. Some gut microbiota may enhance the peripheral tryptophan level, which can cross BBB, and subsequently increase the tryptophan level in CNS, and finally increase the 5-HT level in CNS. In terms of the connection of 5-HT and PD, Tong et al. found that plasma 5-HT levels in PD patients were lower than those in healthy control, and 5-HT levels were negatively correlated with the severity of non-motor symptoms like depression and pain (Tong et al., 2015). Kotagal et al. found that 5-HT may change Aβ metabolism and reduce the risk of PD related cognitive impairment (Kotagal et al., 2018). Lelieveld et al. reported that 5-HT may influence the sleep disordered breathing in PD (Lelieveld et al., 2012). These findings seem to imply the potential associations between 5-HT and the non-motor symptoms in PD, however, require further verification.

###### 2.3.1.1.3 The roles of tryptophan metabolites SCFAs

SCFAs, such as acetic, propionic, butyric, isobutyric, valeric, and isovaleric are commonly produced by gut microorganisms from fermentation of dietary fiber by gut microorganisms (Cummings et al., 1987; Bergman, 1990; Miller and Wolin, 1996). It was documented that *Bifidobacterium, Clostridia, Lactobacillus, Bacteroides,* and *Faecalibacterium* can produce SCFA (Hold et al., 2003; Chambers et al., 2015; Koh et al., 2016; Yang et al., 2020). SCFAs, serve as the primary nutrients of the colonocyte, play a fundamental role in maintaining intestinal homeostasis and regulating energy metabolism (Cummings and Macfarlane, 1997; Priyadarshini et al., 2018). Recently, the SCFAs were proposed as key mediators of microbiota-gut-brain interactions (Dalile et al., 2019). SCFAs are commonly produced in the gut. They can cross BBB, thereby can regulator the inflammation reactions in both periphery and CNS. Peripherally, SCFAs contribute to regulating intestinal permeability and enhancing the integrity of intestinal barrier. Kim et al. found that SCFAs activate G protein coupled receptor (GPR) 41 and GPR43 on intestinal epithelial cells, lead to the production of chemokines and cytokines, and mediate protective immunity and tissue inflammation in mice (Kim et al., 2013). Butyrate administration can affect the expression of tight junction proteins thereby maintain the integrity of intestinal barrier (Plöger et al., 2012; Wang et al., 2012). Additionally, butyrate ministration can increase the levels of tight junction protein and improve intestinal permeability via GPR109A (a G-protein coupled receptor) (Karunaratne et al., 2020). In CNS, SCFAs display the analogous protective effects on BBB. Braniste et al. reported that administration of Clostridium butyricum (mainly produces butyrate) and Bacteroides thetaiotaomicron (mainly produces acetate and propionate) resulted in decrease of the BBB permeability in the frontal cortex, hippocampus, and striatum, along with upregulation of the occludin expression in GF mice, indicating a protective effect on BBB (Braniste et al., 2014). In addition, SCFAs interacting with immune cells, contribute to reduction of the systemic inflammation, subsequently alleviation of the neuroinflammation (Segerstrom and Miller, 2004; Miller and Raison, 2016; Parada Venegas et al., 2019). Moreover, SCFAs constantly modulate maturation and function of microglia in CNS (Smith et al., 2013; Borre et al., 2014). These findings suggest a neuroprotective effect of SCFAs.

Numerous studies elucidated the effects of SCFAs on PD. Aho et al. found the SCFAs levels in PD patients were lower in comparison with healthy people, which were associated with gastrointestinal inflammation and the development of PD (Aho et al., 2021). Another study found that anti-inflammatory butyrate-producing bacteria such as genera *Blautia, Coprococcus*, and *Roseburia* were lower, whereas pro-inflammatory Proteobacteria of the genus *Ralstonia* were higher in the sigmoid mucosa of the PD patients (vs healthy controls). They therefore verified the involvement of pro-inflammatory dysbiosis of gut microbiota in the PD pathophysiology, particularly in the inflammation-induced α-syn misfolding (Keshavarzian et al., 2015). These human results seem to imply the neuroprotective effects of SCFAs on PD, which were also verified by several *in vitro* studies (Chen et al., 2006; Sechi et al., 2008; Fu et al., 2015; Jang et al., 2017). Evidence from the current animal studies also proved the significant effects of SCFAs on PD subjects. However, the results are controversial among studies. Hou et al. found that administration of sodium butyrate achieved suppression of the neuroinflammation and alleviation of the neurological deficits in 1-Methyl-4-phenyl-1,2,3,6-tetrahydropyridine (MPTP) treated mouse PD models (Hou et al., 2021a). Later, they also found that oral administration of gut microbiota-derived propionate resulted in amelioration of the motor deficits and depletion of the dopamine neurons in 6-hydroxydopamine (6-OHDA) induced mouse PD models (Hou et al., 2021b). Conversely, Cannon et al. found the enhancement of butyric acid level and reduction of isobutyric acid level in a Pink1−/- PD mouse (Cannon et al., 2020). Qiao et al. found that administration of sodium butyrate resulted in deterioration of the motor deficits and neuroinflammatory by increasing the number of microglia and activated astrocytes (Qiao et al., 2020). These contradictory results imply an extremely complex nature of SCFAs under a PD condition. The problem whether SCFAs play a neuroprotective or neurotoxic role remains still requires further investigation.

###### 2.3.1.1.4 The roles of tryptophan metabolites BAs

BAs are synthesized by the hepatic cells and secreted to the intestine through the bile duct. Primary BAs are transformed into secondary BAs under the action of gut microbiota in the terminal ileum and colon (Russell and Setchell, 1992; Ridlon et al., 2006). BAs can cross the BBB. It has been reported that BAs are distributed in the brain in human and animals, of which approximately 20 BAs were identified in the rat brain (Xie et al., 2013; Zheng et al., 2016; Pan et al., 2017). Chenodeoxycholic acid (CDCA) and cholic acid (CA) are the major BAs in the rat’s brain (Mano et al., 2004; Zheng et al., 2016). BAs can affect the immune regulation in CNS (Fiorucci et al., 2010). Currently, many studies proved the potential neuroprotective effects of BAs in several neurological diseases. Many studies have demonstrated the potential neuroprotective effects of BAs in various neurological diseases. Furthermore, BAs have been found to be involved in cognitive functions such as learning and memory (Abrams et al., 2006). For instance, intracerebroventricular administration of tauroursodeoxycholic acid (TUDCA) in adult rats significantly enhanced early neurogenesis (Soares et al., 2018). In a mouse model of PD, TUDCA prevented JNK phosphorylation in the midbrain and striatum by regulating Akt signaling and prevented MPTP-induced dopaminergic cell death (Castro-Caldas et al., 2012). Additionally, TUDCA was found to activate Nrf2 to prevent oxidative stress induced by 1-methyl-4-phenylpyridinium and α-syn in an *in vitro* experiment using SH-SY5Y cells (Moreira et al., 2017). TUDCA was also reported to induce mitochondrial autophagy by increasing the expression of PINK1, parkin, and LC3-II/LC3-I in another experiment (Rosa et al., 2017). In a transgenic mouse model of PD, TUDCA reduced striatal cell apoptosis and improved locomotor ability (Keene et al., 2002). Interestingly, TUDCA administration improved cognitive impairment in db/db mice (Liu et al., 2020). Moreover, UDCA was found to have anti-inflammatory potential in the rotenone model of PD, reducing rotenone-induced NF-κB expression and TNF-α levels (Abdelkader et al., 2016). In the study of major depressive disorder, activating the bile acid receptor FXR (farnesoid X receptor) was reported to regulate neuroimmunity and inhibit brain inflammation (Bao et al., 2021). The latest research shows that obeticholic acid (OCA), a derivative of BAs, reduced the expression of neuroinflammatory microglia and IL-1 in the hippocampus (Wu et al., 2021b). Several studies have suggested that BAs may play a role in cognitive dysfunction (Chen et al., 2020b; Jena et al., 2020). In summary, BAs may exert their effects on PD through anti-inflammatory and neuroimmunomodulatory mechanisms.

It is known that BAs and gut microbiota affect mutually. Commonly, BAs are modified by the microorganisms via certain enzymatic reactions. It is easy to understand that the abnormal state of gut microbiota in PD context may affect the modification of BAs. However, the direct evidence remains insufficient. Previous studies reported that several bile salt hydrolase-containing bacteria, such as *Bacteroides* spp.*, Bifidobacterium, and Lactobacillus*, were enhanced in PD patients (Spichak et al., 2021; Ghezzi et al., 2022), indicating a complicated association among PD, microbiota, and BAs (Kiriyama and Nochi, 2023), which is still unclear. Wang et al. documented that BAs might play a role as a “star molecule” in the mechanisms of microbiota-gut-brain axis, which involved in the interactions between gut microbiota and PD (Wang et al., 2023). (5) The roles of tryptophan metabolites γ- Aminobutyric acid.

GABA (γ-Aminobutyric acid), also known as 4-aminobutanoic acid, is a non-protein amino acid and the primary inhibitory neurotransmitter in the mammalian central nervous system(Hyland and Cryan, 2010; Vithlani et al., 2011; Muthukumar et al., 2014). Besides the brain tissue, GABA is present in peripheral tissues such as the heart, stomach, small intestine, liver, and kidney (Akinci and Schofield, 1999; Lakhani et al., 2014). GABA possesses antioxidant, anticonvulsant, anti-inflammatory, analgesic, sedative, and anxiolytic properties (Rorsman et al., 1989; Adeghate and Ponery, 2002; Lacerda et al., 2003; Menezes and Fontes, 2007; Ben-Othman et al., 2017), and has been reported to enhance brain and nervous system function, as well as cognitive and memory functions (Yamatsu et al., 2016; Kim et al., 2019a; Inotsuka et al., 2021). Recent studies have found that GABA produced by gut microbiota (*Bacteroides, Parabacteroides, Escherichia species, Lactobacilli*, and *Bifidobacteria*) can participate in neural activities through the microbiota-gut-brain axis (Yunes et al., 2016; Strandwitz et al., 2019). An animal experiment using probiotics found that *L. rhamnosus (JB-1)* can induce region-dependent changes in GABA mRNA expression in the brain through the vagus nerve, and reduce stress-induced corticosterone and anxiety- and depression-related behavior (Bravo et al., 2011). GF mice were found to have significantly reduced levels of GABA in feces and blood, and antibiotics were found to change the level of GABA in feces, suggesting that the microbiota may contribute to the circulating level of GABA (Matsumoto et al., 2017; Fujisaka et al., 2018). A study on *Caenorhabditis elegans* found that GABA produced by Escherichia coli HT115 has a protective effect on neurons (Urrutia et al., 2020). while a previous study on learning in bees reported that the brains of bees colonized with the heritable *Bifidobacterium* strain displayed an increased level of GABA (Wu et al., 2021a). It has been reported in the literature that GABA plays a crucial role in protecting the intestinal barrier and preventing gastrointestinal mucosal inflammation (Xie et al., 2017; Chen et al., 2019). GABA may contribute to regulation of the population and diversity of gut microbiota, such as enhancement of the dominant microorganism populations, enrichment of the microbial community (Chen et al., 2019). In addition, GABA may improve the colon health by enhancement of the levels of acetate, propionate, butyrate and total short-chain fatty acids, along with decrease of colonic and cecal pH values (Xie et al., 2017). Recent clinical trials and animal studies have shown that GABA plays an important role in the pathogenesis of PD. An experiment examining the GABA levels of PD patients and healthy controls found that the GABA levels in the upper brainstem of PD patients were significantly lower than those of healthy controls (Song et al., 2021b). while a preliminary study also found that a single dose of L-dopa increases upper brainstem GABA in PD (Song et al., 2021c). Some experts suggest that the reduced GABA levels in PD patients may be associated with GABAergic dysfunction (Elmaki et al., 2018). Animal experiments have also reported that polymannuronic acid regulates GABA levels through the microbiota-gut-brain axis in PD mice to prevent the loss of dopaminergic neurons (Dong et al., 2020). Based on the aforementioned information, we can hypothesize that GABA plays an important role in the pathogenesis of PD mediated by the microbiota-gut-brain axis.

##### 2.3.1.2 The roles of LPS

LPS, also known as endotoxin, is an integral structural component of the outer membrane of gram-negative bacteria (Raetz and Whitfield, 2002). Dysbiosis of the microbiota leads to increased intestinal permeability, allowing LPS products to translocate from the intestinal lumen into the host circulation (Rietschel et al., 1994; Tilg et al., 2016). Several studies have found a significant increase in gram-negative bacteria in PD patients, which is closely related to intestinal inflammation (Guo et al., 2015; Nighot et al., 2017; Gorecki et al., 2019). Furthermore, a study found a positive association between the relative abundance of gram-negative Enterobacteriaceae and the severity of postural instability and gait difficulty (Scheperjans et al., 2015). An animal experiment demonstrated that injecting LPS into the rat substantia nigra can activate microglia, trigger the inflammatory response, increase the expression of pro-inflammatory cytokines, and change the activity of oxidative stress markers, inducing the characterization of a PD model (Sharma and Nehru, 2015). Different routes and modes of LPS administration can cause various parkinsonian symptoms. Intraperitoneal injection of LPS can increase α-Syn expression in the large intestine and intestinal permeability (Kelly et al., 2014). The PD model induced by endotoxin can be established by unilateral intranasal drip of LPS. These mice showed progressive hypokinesia, selective loss of dopaminergic neurons (He et al., 2013), decreased dopamine (DA) content in the striatum, and α-Syn aggregates without systemic inflammation and immune response (He et al., 2013). Intra-rectal injection of LPS derived from P. mirabilis can induce inflammation in the nigrostriatal regions of the brain (Choi et al., 2018). Intestinal biopsy of PD patients revealed a close relationship between increased intestinal permeability and intestinal α-syn and LPS (Forsyth et al., 2011). Moreover, a series of *in vitro* and *in vivo* experiments proved the relationship between LPS and intestinal permeability and the associated inflammatory signals (Guo et al., 2013; Nighot et al., 2017; Nighot et al., 2019). Previous research has shown that inflammatory monocytes primed with LPS can internalize α-syn, which in turn facilitates its dissemination from the periphery towards the brain and spinal cord (Peralta Ramos et al., 2019). In conclusion, it is speculated that LPS releases inflammatory factors and induces α-syn accumulation in the intestine, which then reaches the brain through the circulatory system, resulting in PD-related pathological changes.

##### 2.3.1.3 The roles of hydrogen

Hydrogen is a metabolite of bacterial fermentation in the gut and has been shown to have selective antioxidant and anti-inflammatory effects (Levitt, 1969; Ohsawa et al., 2007). Furthermore, it has been found to possess anti-apoptotic and cytoprotective properties, contributing significantly to cell protection (Xie et al., 2010; Dixon et al., 2013). In a rat model of PD, treatment with hydrogen was reportedly neuroprotective and prevented dopaminergic cell loss (Fu et al., 2009). Mice models and PD patients also showed similar findings (Fujita et al., 2009; Yoritaka et al., 2013). An animal experiment demonstrated that drinking hydrogen water and intermittent hydrogen exposure can prevent 6-hydroxydopamine-induced PD in rats (Ito et al., 2012). Moreover, evidence has shown that the generation and oxidation of H2 help maintain the diversity of the gut microbiome (Hylemon et al., 2018). A study discovered that probiotics can promote the intestinal production of hydrogen and improve metabolic syndrome in C57BL/6J mice (Zhao et al., 2019). Analysis of 16S rRNA gene isolates indicated a low abundance of hydrogen-producing bacteria in PD patients (Hasegawa et al., 2015). Gas chromatography measurements showed that compared to the control group, the intestinal hydrogen production of PD patients was 2.2 times lower (Suzuki et al., 2018). A clinical trial found that oral intake of lactulose can increase the concentration of hydrogen in PD patients (Ito et al., 2012). Subsequent studies provided evidence that lactulose may increase hydrogen production by the intestinal microbiota (Ohno et al., 2012). These findings suggest that the abundance of intestinal hydrogen-producing bacteria may be related to the occurrence and development of PD. Therefore, intestinal-derived hydrogen may serve as a novel candidate non-invasive biomarker for diagnosis, prognosis, and treatment response in PD.

#### 2.3.2 Immune systems pathway

It is known that the immune system, no matter innate immune system and adaptive immune system, is closely associated with the gut microbiota. Gut microbiota plays a key role in maintaining intestinal mucosal barrier and contributes to maintenance and development of the immune system (Fernández et al., 2003; Candela et al., 2008; Olszak et al., 2012). Immune function of gut microbiota initiates in the intestine and then regulates systemic immunity during the immune response (Dai et al., 2020). A number of early studies documented that implantation of segmented filamentous bacteria from the small intestine resulted in recovery of the immune function of intestinal B and T lymphocytes (Umesaki et al., 1995; Talham et al., 1999; Umesaki et al., 1999). Later, Forsythe found that implantation of the intestinal microorganism achieved reconstruction of the immune function, which is deficient in a GF mouse (Forsythe et al., 2010). Nevertheless, immunoreactions in CNS and the peripheral organs are relatively independent under the condition that BBB is unbroken. Due to aforementioned BBB state in PD, here, we only discuss the inherent cellular immune pathway in CNS.

##### 2.3.2.1 Microglia

Microglia are key immune cells in the CNS, which contribute to presentation of antigen, production of cytokines, and activation/regulation of inflammation (Chen et al., 2020a; Jiang et al., 2020). Numerous studies have elucidated that microglia are remarkably affected by changes of gut microbiota. Erny et al. found that the proportions of immature microglia in GF mice increased in GF mice. Interestingly, once the gut microbiota in these GF mice was recovered, the features of microglia were accordingly recovered (Erny et al., 2015). Alternatively, knockout of the microglia-specific gene MafB upregulated the expression of the inflammatory pathways in adult GF mice (Matcovitch-Natan et al., 2016). Song et al. found that dysbiosis of gut microbiota caused suppression of the microglia activation and upregulation of the TLR4/NF-κB inflammatory pathway in the hippocampus of aging rats (Song et al., 2021a). Accordingly, treatment of FMT can alleviate the dysbiosis of gut microbiota and resulted in suppression of the microglia activation (Sun et al., 2018). These results reveal a close interaction between microglia and gut microbiota, even the aforementioned experiments were using the animals which BBB seems to be unbroken. Many authors believed that the bidirectional interactions between microglia and gut microbiota might play a role in the etiology of neurodegenerative diseases (Balducci and Forloni, 2018; Davoli-Ferreira et al., 2021; Wang et al., 2021b; Zheng et al., 2023). In the PD scenario, this hypothesis is also supported by many experiments. Sampson et al. proved that gut microbiota plays a role in the motor deficits, microglia activation and α-syn pathology in a α-syn overexpressed mouse (Sampson et al., 2016). Fang et al. found that using the mouse strain MG1363-pMG36e-GLP-1 expressing glucagon-like peptide-1 to treat MPTP-treated mice resulted in amelioration of the motor deficits, suppression of the microglia activation and inflammation, reduction of the pathogen Enterobacteriaceae, and enhancement of the probiotics Lactobacillus and Akkermansia (Fang et al., 2019). Joers et al. treated the PD monkey models with a novel TNF inhibitor, XPro1595. They found that the diversity of gut microbiota, inflammatory, and the microglial behavior were correspondingly changed (Joers et al., 2020). Sun et al. found that oral administration of Clostridium butyricum achieved improvement of the motor deficits, dopamine neuron depletion, synaptic dysfunction and microglia activation in MPTP treated mice (Sun et al., 2021a). All these experiments verified the close relationship between microglia and gut microbiota under the PD condition from various treatments and animal models. Hence, Claudino Dos Santos et al. recently commented that inflammatory reactions may activate microglia and induce aggregation of α-syn, ultimately cause damage of dopaminergic neurons, where dysbiosis of gut microbiota plays a key role (Claudino Dos Santos et al., 2023). There is no clinical evidence investigating the roles of microglia during the intervention of gut microbiota in PD patients. Importantly, there is no study observing changes of the gut microbiota once the microglia in PD are intervened. The complicated interactions among microglia, PD, and gut microbiota are still intricate, which require further investigation in the future.

##### 2.3.2.2 Astrocytes

As the most abundant cells in CNS, astrocytes also play a role in the regulation of the inflammatory and immune reactivity. Interactions between astrocytes and gut microbiota are also reported. Rothhammer et al. reported that dietary tryptophan is metabolized into aryl hydrocarbon receptor agonists by the gut microbiota, and then act on astrocytes to suppress CNS inflammation (Rothhammer et al., 2016). Zhang et al. found that FMT from the NLRP3 KO gut microbiota significantly ameliorated the depression-like behaviors along with astrocyte dysfunction via suppression of the circular RNA HIPK2 (circHIPK2) expression in animal depression models. They hence proposed a gut microbiota-circHIPK2-astrocyte axis in depression (Zhang et al., 2019). Sanmarco et al. found an astrocyte subset, LAMP1+TRAIL+ astrocytes suppressed the CNS inflammation by inducing T cell apoptosis, while these LAMP1+TRAIL+ astrocytes are maintained by the microbiome-licensed meningeal IFN+ NK cells (Sanmarco et al., 2021). Margineanu et al. reported that the hippocampal expression of astrocyte-neuron lactate shuttle related genes was regulated by the gut microbiota (Margineanu et al., 2020). Another study indicated that greater level of gut microbiome-derived metabolite trimethylamine N-oxide was associated with higher expression of brain pro-inflammatory cytokines and astrocyte activation markers (Brunt et al., 2021). These studies also elucidate the crucial role of astrocytes in the immune modulation in the CNS. In terms of PD, Sun et al. found that FMT protected the neuronal function by inhibition of the neuroinflammation and reduction of astrocytes activation in the substantia nigra (Sun et al., 2018). Another acupunctural study found that acupuncture on PD mice regulated the abundance of gut microbiota and restored the overexpression of astrocytes in striatum and substantia nigra (Jang et al., 2020). By far, the evidence between astrocytes and gut microbiota in PD remains limited.

##### 2.3.2.3 Inflammatory mediators

To the extent known, the modulations between PD and dysbiosis of the gut microbiota are bidirectional. *i)* Dysbiosis of gut microbiota may cause PD-related abnormal inflammatory response. Gut microbiota may regulate immune-related inflammatory factors in the intestine, and subsequently affect the CNS (Shimizu et al., 2011; Huang et al., 2019c; Claudino Dos Santos et al., 2023). Microbiota along with their products can stimulate intestinal epithelial cells and macrophages in the gut, resulting in activation of immune response and the release of inflammatory cytokines, and they can reach the CNS (Banks, 2009; Mayer et al., 2014; Gorky and Schwaber, 2016). At autopsy, it was found that the expression of pro-inflammatory cytokines and chemokines were upregulated in the brain tissue and cerebrospinal fluid of patients with PD, including TNF-α, IL-1β, IL-6 and IFN-γ (Mogi et al., 1994a; Mogi et al., 1994b). Knockout of the TNF-α receptor gene exhibited neuroprotective effects in mice (Bhaskar et al., 2014). Under the physiological conditions, gut microbiota downregulates IFN-γ expression in astrocytes while upregulates TRAIL expression in astrocytes (Sanmarco et al., 2021). The gut microbiota can produce various toll like receptor (TLR) ligands that can play a pro-inflammatory role in a specific environment (Caputi and Giron, 2018). Thus, any changes of gut microbiota and destruction of the gut epithelial barrier may activate TLRs, and promote inflammatory response in both the gut and brain of PD patients (Terán-Ventura et al., 2014; Dheer et al., 2016; Sun et al., 2018). It has been known that the signal regulation of TLR2 and TLR4 can affect the development of PD (Harms et al., 2018; Pascual et al., 2021). In the intestines of PD patients, enhancement of CD3+ T cells in colon tissue was found along with the immune interactions among TLR4, cytokines, and chemokines (Terán-Ventura et al., 2014). Epidemiological studies also suggested that inflammatory bowel disease is a significant risk factor for the development of PD (Lin et al., 2016; Villumsen et al., 2019; Weimers et al., 2019). Some scholars hypothesized that the alteration of gut microbial flora leads to gastrointestinal system disturbance which cause neuroinflammation by prion α-syn expression and produces PD like symptoms (Sharma et al., 2019). *ii)* PD may have GI dysfunction, which worsen the state of gut microbiota. In PD patients at the early stage, mild intestinal inflammation is commonly developed that triggers a low-level immune response and increases intestinal permeability (Houser and Tansey, 2017). Accordingly, intestinal inflammation and enhanced intestinal permeability are the intestinal characteristics of PD patients (Houser and Tansey, 2017; Perez-Pardo et al., 2019). These issues markedly break the balance of gut microbiota and deteriorate their ecology. Thus, the imbalance of intestinal microecology increases the pro-inflammatory environment in the intestine, which promotes the neuroinflammation of the central nervous system and leads to the development of PD. In this regard, PD and dysbiosis of the gut microbiota may generate a bidirectional vicious circle, and promote the development and progression of PD.

It has been reported that α-syn misfolding might initiate in the gut, and spread to the brain via the vagus nerve (Claudino Dos Santos et al., 2023). This hypothesis provided a plausible “pathway” of the impacts of gut microbiota to the α-syn-based PD pathogenesis (see next section). However, due to the complex nature of interactions among microglia, astrocytes, gut microbiota and enteric glia, we believe that the actual interactions between PD and gut microbiota -related inflammatory reactions are far more complex than “bidirectional”. It might be “multidirectional” involved by many known and unknown factors, which indeed warrant further investigation.

#### 2.3.3 Neuronal pathways

##### 2.3.3.1 The vagus nerve pathway

The vagus nerve, comprising the tenth pair of cranial nerves, is the longest and most widely distributed pair of cranial nerves, containing sensory, motor, and parasympathetic nerve fibers (Rosas-Ballina et al., 2011; McVey Neufeld et al., 2019; van der Meij and Wermer, 2021). It serves as a crucial link between gut bacteria and the brain, transmitting signals from various intestinal tracts and being associated with numerous gastrointestinal tracts, nervous system, and immune system diseases (Berthoud and Neuhuber, 2000; Goehler et al., 2000; Powley et al., 2011; Ahmed et al., 2019). The vagus nerve communicates with the brain through multiple synaptic connections with the nucleus tractus solitarius of the brain stem (Biaggioni, 2017). According to reports, when gut microbiota stimulate the afferent neurons of ENS, the ENS and vagus nerve form a synaptic connection and an information transmission pathway, enabling mutual communication and regulation between gut microbiota and the brain (Yu et al., 2020). Studies have also found that sensory neurons of the vagus nerve form various mechanical and chemical sensory terminals along the gastrointestinal tract to receive intestinal-brain signals (Bravo et al., 2011; Perez-Burgos et al., 2013). Additionally, enteroendocrine cells can form synapses with neighboring nerves to assist the vagus nerve in receiving signals from the intestine (Bohórquez et al., 2015; Bellono et al., 2017; Fülling et al., 2019). A large prospective cohort study found that subjects who have undergone complete vagotomy have a low risk of PD (Svensson et al., 2015). A post-mortem study has shown that PD pathology may start in the gastrointestinal tract and then spread through the vagus nerve to the brain (Braak et al., 2006). An animal study found that the central role of Lactobacillus rhamnosus was eliminated after vagus nerve amputation (Fung et al., 2017). Recently, α-syn has emerged as a critical factor contributing to PD pathogenesis (Mor et al., 2017). More evidence has indicated that the vagus nerve is a route by which α-syn pathology can spread both to and from the brain (Pan-Montojo et al., 2012; Matteoli and Boeckxstaens, 2013; Gray et al., 2014; Liu et al., 2017). A new mouse model of PD demonstrates α-syn pathology spreading from the gut to the brain via the vagus nerve (Kim et al., 2019b). Recent advances have suggested that gut bacteria enhance the aggregation of α-syn and other similar proteins, a mechanism that might promote the ability of pathogens to reach the CNS through the vagus nerve (Sampson et al., 2016; Choudhry and Perlmuter, 2017). However, some scholars denied the connection between PD and the vagus nerve (Tysnes et al., 2015). A study using baboons as a model found a possible systemic mechanism in which the general circulation would act as a route for long-distance bidirectional transmission of endogenous α-syn between the enteric and the central nervous systems (Arotcarena et al., 2020). Although accumulated and convincing evidence supports this pathway, its specific mechanisms remains unclear and requires further exploration.

##### 2.3.3.2 The HPA axis pathway

The HPA axis is a crucial component of the neuroendocrine system that regulates various body processes to cope with stress (Smith and Vale, 2006). Under stress, Corticotropin-releasing factor (CRF) is released from the paraventricular nucleus (PVN) of the hypothalamus, which is the main regulator of the HPA axis and induces the release of adrenocorticotropic hormone (ACTH) into systemic circulation (Mastorakos and Zapanti, 2004). Subsequently, the adrenal cortex is stimulated by ACTH and secretes glucocorticoids (Smith and Vale, 2006). The released glucocorticoids then bind to glucocorticoid receptors (GR), resulting in feedback inhibition (Keller-Wood and Dallman, 1984). In recent years, more and more studies have been conducted to discover a bidirectional communication between the neuroendocrine system and gut microbiota through the HPA axis (Farzi et al., 2018). The CNS can regulate intestinal function through the HPA axis, and the gut microbiota can also regulate gut hormone levels, which affect CNS function through the HPA axis (Sun et al., 2020). An animal experiment using GF mice found that Enterococcus faecalis can inhibit excessive stress response in social interaction by limiting the production of corticosterone levels mediated by the HPA axis, thereby promoting the social activity of mice (Wu et al., 2021c). An experiment using GF and pathogen-free mice demonstrated that maternal isolated early life stress altered the HPA axis in a microbiota-independent fashion (Jiang et al., 2015; Kelly et al., 2016). Additionally, evidence suggests that GF mice can overstimulate the HPA axis under stress, while probiotics can alleviate the response state of the HPA axis in mice (Heym et al., 2019; Huang et al., 2019b). A study from 2013 found that DBS of the subthalamic nucleus can reduce cortisol levels in patients with advanced PD (Seifried et al., 2013). Psychological stress and changes in the hypothalamic-pituitary-adrenal axis were also present in patients with “*de novo*” PD (Ibrahimagic et al., 2016). Increasing numbers of clinical trials and animal experiments showed that the HPA axis was altered and unbalanced in PD patients, leading to a significant increase in cortisol levels (Höschl and Hajek, 2001; Zhang et al., 2017; Grigoruţă et al., 2020). Interestingly, one study found that non-motor symptoms of PD (e.g., mood and anxiety) were significantly associated with hair cortisone levels (van den Heuvel et al., 2020). Hair glucocorticoid levels reflect longer-term HPA-axis function and can provide additional insights into the role of a dysregulated HPA-axis in PD (Wosu et al., 2013; Stalder et al., 2017). In conclusion, although there are few studies on the role of the HPA axis in gut microbiota and PD, we cannot ignore the roles of the HPA axis in the microbiota-gut-brain axis of PD.

## 3 Treatment of PD Targeted at Gut Microbiota

In the clinical setting, the primary treatment for PD remains dopaminergic medication. Currently, there is no known cure for PD. Although growing evidence supporting the role of gut microbiota in PD, the gut microbiota-based therapy is still under investigation. This section summarizes the emerging preventive and therapeutic interventions that modulate the gut microbiome (**Table 3**).

**Table 3:** Documented gut microbiota-related PD treatments.

Therapeutic method	Names	Research subjects	Changes in intestinal microflora, metabolites, or related factors		Principal results
			Increased abundance	Decreased abundance	
Prebiotics	GOS (Savignac et al., 2013)	Adult male Sprague Dawley rats	Bifidobacteria	Not listed	Elevated central BDNF, NMDAR and D-serine
	BGOS (Williams et al., 2016)	Neonatal rats	Not listed		Increased the levels of synaptophysin, GluN2A-subunits and BDNF proteins
	Xyloolidosaccharide (Chunchai et al., 2018)	High-fat-diet mice	Decreased plasma insulin level, HOMA index, area under the curve of the oral glucose tolerance test, plasma total cholesterol level, and LDL cholesterol level		Restored the cognitive function
	Lactulose (Yuan et al., 2021)	Mice after stroke	Bradyrhizobium, Oceanobacillus, Escherichia, and Leptothrix	Lactobacillus, Clostridium, Flavobacterium, Brachybacterium, and Helicobacter	Repaired intestinal barrier injuries, improved gut microbiota imbalances, and reduced post-stroke inflammatory responses
	Fermented milk containing prebiotic fiber (Barichella et al., 2016)	Patients with PD with Rome III–confirmed constipation	Resulted in a higher increase in the number of total complete bowel movements and in stool consistency, as well as in a larger reduction in the use of laxatives.		Improved constipation in patients with PD
	Prebiotic fibers (Perez-Pardo et al., 2017)	The rotenone PD model	Improved rotenone-induced delayed intestinal transit and reduced rotenone-induced alpha-synuclein and GFAP overexpression in the colon.		Normalized rotenone-induced motor and non-motor abnormalities
Probiotics	Milk drink containing 6.5×109 CFU of Lactobacilus casei Shirota (Cassani et al., 2011)	Patients with PD	Increased in the number of days per week in which stools were of normal consistency and significant reductions in the number of days per week in which patients felt bloated, experienced abdominal pain and sensation of incomplete emptying		Improved stool consistency and defecation habits
	A mixture of probiotics (Tamtaji et al., 2019)	Patients with PD	Decreased movement disorders society-unified parkinson’s disease rating scale, reduced high-sensitivity C-reactive protein and malondialdehyde, and enhanced glutathione levels.		Improved the clinical symptoms and reduced inflammation and oxidative response
	Actobacillus and bifidobacterium (Magistrelli et al., 2019)	PBMCs isolated from PD patients	Reduced pro-inflammatory and increased the anti-inflammatory cytokines		Decreased pro-inflammatory cytokines, oxidative stress
	The probiotics consisted of six bacterial strains (Hsieh et al., 2020)	A genetic MitoPark PD mouse model	Reduced the motor impairments in gait pattern, balance function, and motor coordination.		Reduced the deterioration of motor dysfunction
	Bifidobacterium longum BB536 and Lactobacillus rhamnosus HN001(Ilie et al., 2021)	A rotenone-induced PD zebrafish model	An increase of distance swam in the first days of the treatment with a highest value		No statistical significance
	A mixture of probiotics (Alipour Nosrani et al., 2021)	6-hydroxydopamine induced PD rat models	Improved rotation behavior, cognitive function, lipid peroxidation, and neuronal damage		Probiotics had neuroprotective effects
	A mixture of probiotics (Srivastav et al., 2019)	PD models induced by MPTP or rotenone	Downregulated expression of monoamine oxidase B in the striatum		Ameliorated neurodegeneration
	SLAB51 (Castelli et al., 2020)	6-hydroxydopamine induced PD rat models	Modulated the BNDF pathway, increased neuroprotective protein levels and decreased the neuronal death proteins		Confirmed a neuroprotective effect exerted by the probiotics.
	Bacillus subtilis (Goya et al., 2020)	Caenorhabditis elegans model of synucleinopathy	Inhibited α-syn aggregation and clears preformed aggregates in an established Caenorhabditis elegans model of synucleinopathy		Protected against α-syn Aggregation in C. elegans
FMT	Fecal bacterial liquid from healthy donors (Kuai et al., 2021)	Patients with PD	*Blautia* and *Prevotella*	*Bacteroidetes*	The intestinal bacterial overgrowth in PD patients returned to normal
	Fecal bacterial liquid from healthy donors (Huang et al., 2019a)	A PD patient	*Lachnoclostridium,* Dialister, Alistipes, and Unidentified-Ruminococcaceae	*Blautia*, *Coprococcus,* and *Roseburia*	The constipation and tremors were effectively relieved
	Fecal bacterial liquid from healthy donors (Xue et al., 2020)	Patients with PD	Relieved the motor and non-motor symptoms with acceptable safety in PD		Compared with nasointestinal FMT, colonic FMT may be more effective
	Fecal bacteria from healthy mice (Sun et al., 2018)	MPTP-induced PD mice	Firmicutes	Proteobacteria	Protected PD mice by suppressing neuroinflammation and reducing TLR4/TNF-α signaling.

### 3.1 Prebiotics

Prebiotics are organic substances that are not digested and absorbed by the host, but selectively promote the metabolism and proliferation of beneficial bacteria in the body, thereby improving host health. Once they reach the colon, prebiotics are decomposed and utilized, promoting the growth of colonic microbiota, where they play an important role in improving the intestinal microecology and regulating lipid metabolism. Different types of prebiotics include isomalto-oligosaccharide, fructooligosaccharide, galacto-oligosaccharide (GOS), xylooligosaccharide, lactulooligosaccharide, soybean oligosaccharide, and inulin (Slavin, 2013). Although there are only few studies using prebiotics in the treatment of PD, neuroprotective effects of prebiotics were verified. Feeding GOS to rats could elevate the expression of central brain-derived neurotrophic factor (BDNF), N-methyl-D-aspartate receptor subunit (NMDAR), and D-serine (Savignac et al., 2013). In another experiment, neonatal prebiotic (BGOS) supplementation increased the levels of synaptophysin, GluN2A-subunits, and BDNF proteins in the adult rat hippocampus (Williams et al., 2016). Additionally, it was reported that xylooligosaccharide could effectively restore the cognitive function of obese-insulin resistant rats by reducing the activation of microglia through the gut-brain axis (Chunchai et al., 2018). Lactulose was also found to contribute to repairing intestinal barrier injuries, improving gut microbiota imbalances, and reducing post-stroke inflammatory responses in the stroke mice (Yuan et al., 2021). Findings of these studies demonstrated that prebiotics may play an important role in the improvement of neurological functions. Moreover, prebiotics could improve immune function and regulate bowel defecation habits (Jeurink et al., 2013; Meksawan et al., 2016). We speculate that this may be associated with amelioration of the inflammation and gastrointestinal dysfunction in PD. A clinical trial found that a fermented milk containing prebiotic fiber was superior to placebo in improving constipation in PD patients (Barichella et al., 2016). In PD striatum rotenone model mice, prebiotic fibers were found to partially alleviate the motor and non-motor problems caused by rotenone (Perez-Pardo et al., 2017). Additionally, it restored the level of striatal dopamine transporter in PD mice, indicating its neurorestorative properties (Perez-Pardo et al., 2017). In summary, the prebiotics-related mechanisms might lie in improving the composition of the gut microbiota, improving intestinal barrier function, and showing potential beneficial effects on the regulation of the microbiota-gut-brain axis.

### 3.2 Probiotics

Probiotics are microorganisms that can improve the balance of gut microflora thereby exhibit beneficial effects in the host. The majority of available studies reported that administration of probiotic achieved neuroprotective benefits and alleviation of cognitive impairments (Ji and Shen, 2021). Probiotics can exert beneficial effects via decline of neurotransmitter levels, chronic inflammation, oxidative stress, and apoptosis in NDDs (Westfall et al., 2017). An early study found that regular intake of probiotics significantly improved stool consistency and defecation habits in patients with PD (Cassani et al., 2011). A clinical trial found that a mixture of probiotics (*Lactobacillus acidophilus, Bifidobacterium bifidum, Lactobacillus reuteri, and Lactobacillus fermentum*) ameliorated the clinical symptoms and reduced inflammatory and oxidative responses in PD patients (Tamtaji et al., 2019). *In vitro* experiments found that probiotics (*Lactobacillus and Bifidobacterium*) inhibited the production of inflammatory cytokines and ROS in PD patients (Magistrelli et al., 2019). In line with the clinical studies, it was documented that long-term administration of probiotics achieved protection of dopamine neurons and amelioration of the motor dysfunction in PD mice (Hsieh et al., 2020). In the rat model of PD induced by 6-hydroxydopamine, administration of probiotics could improve the rotational behavior, cognitive function, lipid peroxidation, and neuronal damage (Alipour Nosrani et al., 2021). In another experiment, it was found that a mixture of probiotics could protect dopaminergic neurons against neurotoxic effects induced by MPTP or rotenone exposure (Srivastav et al., 2019). *In vivo* and *in vitro* experiments demonstrated that SLAB51, a novel probiotic agent, could regulate the BDNF pathway, increase the level of neuroprotective proteins, and reduce neuronal death proteins. Thus, the neuroprotective effects of probiotics were verified (Castelli et al., 2020). In addition, probiotics (*Bifidobacterium longum BB536* and *Lactobacillus rhamnosus HN001*) were found to have tendency to improve the swimming performance and reduce the oxidative stress in a rotenone-induced PD zebrafish model (Ilie et al., 2021). Interestingly, the probiotic *Bacillus subtilis* was reportedly to prevent α-syn aggregation in *Caenorhabditis* elegans models (Goya et al., 2020). Accordingly, probiotics can be considered as an emerging treatment for PD subjects.

### 3.3 FMT

FMT refers to transfer of functional bacteria from feces of healthy individuals to the intestinal tracts of patients in a specialized manner to regulate the gut microbiota. The objective of FMT is to restore the healthy diversity of gut microbiota and provide successful therapy for certain diseases both within and outside the intestinal tract. Recently, FMT has also been considered as a potential therapy for the treatment of NDDs, particularly for PD (Sun et al., 2021b; Wang et al., 2021a). Kuai et al. found that the abundance of *Blautia* and *Prevotella* increased after undergoing FMT in PD patients, whereas the abundance of *Bacteroides* decreased sharply, and the PAC-QOL scores and Wexner constipation scores significantly decreased (Kuai et al., 2021). Huang et al. found that constipation and tremors were effectively relieved after undergoing the FMT treatments (Huang et al., 2019a). Additionally, Xue et al. found that FMT alleviated the motor and non-motor symptoms in PD patients, and colonic FMT was more preferable than nasointestinal FMT (Xue et al., 2020). Oral administration of specific microbial metabolites to GF mice promoted neuroinflammation and motor symptoms, meanwhile, colonization of α-syn-overexpressing mice with feces from PD patients deteriorated the physical impairments in animals (Sampson et al., 2016). In MPTP-induced PD mice, FMT was found to achieve reducing dysbiosis of gut microbial, decreasing fecal SCFAs, alleviating physical impairments, and increasing striatal DA and 5-HT levels (Sun et al., 2018). These results suggest that FMT the feces of healthy subjects could change the microecology of the intestine, thereby exhibit therapeutic effects in PD subjects.

## 4 Concluding remarks

The refractory nature of PD treatments, particularly for advanced PD so far, requires better exploration of the novel strategies other than dopaminergic-based treatments. Undoubtedly the gut microbiota-related pathogenesis has been highlighted and become a beacon for the drug development. Although advances in the technologies like high-throughput sequencing, GF animals and GMT make deep investigation of gut microbiota become feasible, the associations between gut microbiota and PD are still intricate due to the complex nature of interactions among gut microbiota as well as the relative influence factors. The present review summarized the potential the gut microbiota-related mechanisms from the perspectives of humoral pathway, cellular immune pathway and neuronal pathway. Numerous studies have elucidated the potential mechanisms from these different angles. It seems a plausible hypothesis that α-syn might initiate in the gut induced by the dysbiosis of gut microbiota, and then transport into CNS with a “prion-like” manner via several pathways (such as the vagus nerve pathway) (Claudino Dos Santos et al., 2023). However, we still believe the association between gut microbiota and PD is far more complex than “bidirectional”, it must be “multidirectional”, which require further investigation. So far, there is still a long way before the gut microbiota-related strategy against PD can be actually application in the clinical setting. Future investigation should focus on the following issues: *i)* Clarification of delivery mechanisms of the gut-original α-syn into brain. How to intervene such delivery processes (prevention of PD)? *ii)* Other than the α-syn-related mechanisms, are there any other PD related pathogenic factors involved in the dysbiosis of gut microbiota (such as KP-related liposoluble small-molecule neurotoxins)? *iii)* Development of gut microbiota-based PD biomarkers with low-cost, low-invasion and high specificity is indispensable. *iv)* Establishment of GF animal models in large animals, such as non-human primates, is needed. *v)* Implementation of well-designed clinical trials of GMT, prebiotics and probiotics. More investigations with comprehensive and rigorous experimental design are therefore highly anticipated.

## Author contributions

XJ: Conceptualization, Formal analysis, Software, Writing – original draft, Writing – review & editing. QC: Funding acquisition, Writing – original draft. YZ: Funding acquisition, Writing – original draft. TA: Conceptualization, Funding acquisition, Writing – review & editing, Supervision.
